# metabolomicsR: a streamlined workflow to analyze metabolomic data in R

**DOI:** 10.1093/bioadv/vbac067

**Published:** 2022-09-16

**Authors:** Xikun Han, Liming Liang

**Affiliations:** Department of Epidemiology, Harvard T H Chan School of Public Health, Boston, MA 02115, USA; Program in Genetic Epidemiology and Statistical Genetics, Harvard T H Chan School of Public Health, Boston, MA 02115, USA; Department of Epidemiology, Harvard T H Chan School of Public Health, Boston, MA 02115, USA; Program in Genetic Epidemiology and Statistical Genetics, Harvard T H Chan School of Public Health, Boston, MA 02115, USA

## Abstract

**Summary:**

metabolomicsR is a streamlined, flexible and user-friendly R package to preprocess, analyze and visualize metabolomic data. metabolomicsR includes comprehensive functionalities for sample and metabolite quality control, outlier detection, missing value imputation, dimensional reduction, batch effect normalization, data integration, regression, metabolite annotation and visualization of data and results. In this application note, we demonstrate the step-by-step use of the main functions from this package.

**Availability and implementation:**

The metabolomicsR package is available via CRAN and GitHub (https://github.com/XikunHan/metabolomicsR/). A step-by-step online tutorial is available at https://xikunhan.github.io/metabolomicsR/docs/articles/Introduction.html.

**Supplementary information:**

Supplementary data are available at *Bioinformatics Advances* online.

## 1 Introduction

Metabolites are small molecules in biofluids and tissues that represent intermediate products of a variety of physiological processes (Johnson *et al.*, 2016). The comprehensive profiling of metabolites based on high-throughput technologies offers a new opportunity for biomarker discovery. With various omics data types, including genomics, epigenetic and proteomics, the identification of dysfunctional metabolites provides new insights into precision medicine for disease risk prediction, diagnosis and therapeutic treatment (Long *et al.*, 2020; Pinu *et al.*, 2019).

**Fig. 1.**
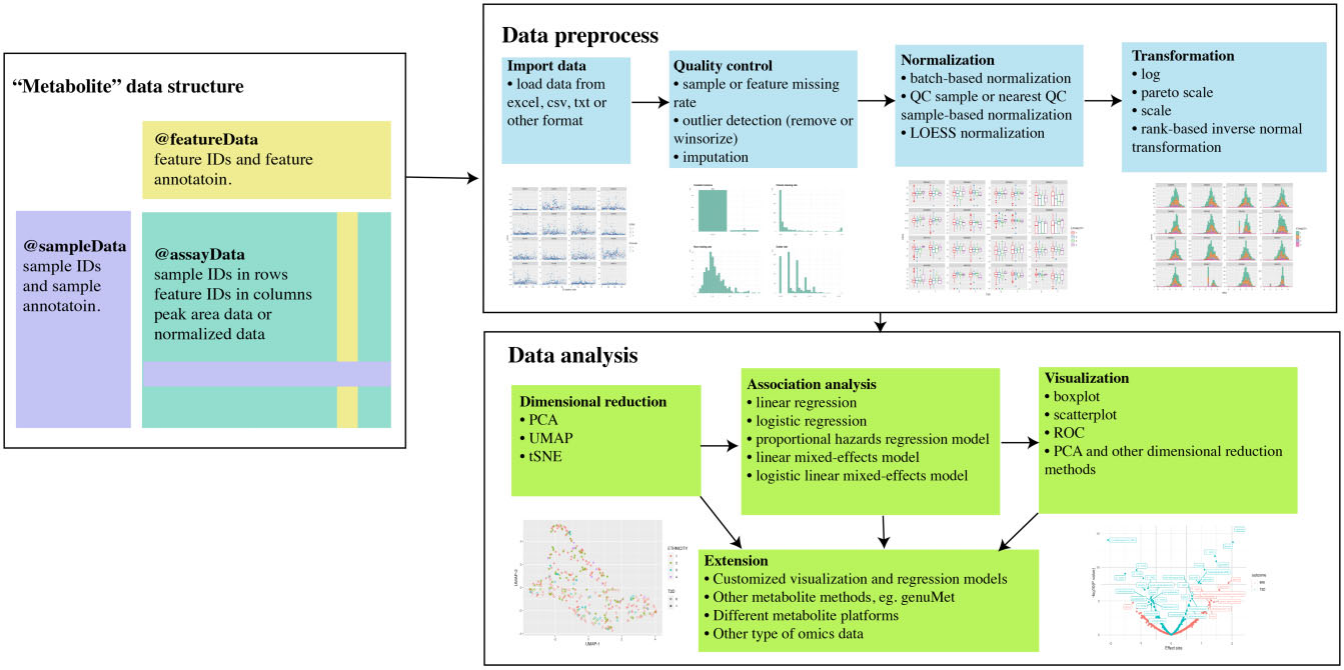
Streamlined workflow to preprocess, analyze and visualize metabolomics data in metabolomicsR. The main functions are categorized in each box. The sub-panel figures are displayed for illustration. The preprocess step includes procedures for quality control, outlier detection, missing value imputation and transformation, etc.

In recent years, several comprehensive packages have been developed for metabolomic data analysis (Supplementary Table S1) (Chetnik *et al.*, 2021; Lloyd *et al.*, 2020; Pang *et al.*, 2021; Stanstrup *et al.*, 2019); however, a streamlined and flexible workflow with one-stop-access to comprehensive functions for population-based metabolomics studies remains needed (Long *et al.*, 2020). For instance, MetaboAnalystR provided various web-based tools (Pang *et al.*, 2021), the structToolbox package provides a suite of complex class templates (Lloyd *et al.*, 2020) and the maplet package (Chetnik *et al.*, 2021) can record all intermediate steps and results; however, a streamlined workflow remains warranted (Supplementary Table S1). In this package, we built a framework for metabolomics data and provided a user-friendly pipeline for the most necessary analytic functions used in population-based metabolomics studies (Fig. 1). Our metabolomicsR package provided an easy-to-use and extensible framework for metabolomic research with a detailed online tutorial.

## 2 metabolomicsR application

The metabolomicsR is designed to be a comprehensive R package that can be easily used by researchers with basic R programming skills. The framework designed here is versatile and extensible to other various methods.

### 2.1 Data structure

We first designed a ‘Metabolite’ class based on the object-oriented programming system S4 in R. For a particular ‘Metabolite’ data, it will include ‘assayData’ (e.g. peak area data before or after QC, samples in rows and metabolites in columns), ‘featureData’ (metabolite annotation), ‘sampleData’ (sample annotation), ‘featureID’, ‘sampleID’, ‘logs’ (log information of data analysis) and ‘miscData’ (other ancillary data).

### 2.2 Import data

To demonstrate the package, we obtained the data from the Qatar Metabolomics Study on Diabetes (Yousri *et al.*, 2015), similar to the data format from non-targeted mass spectrometry by usual research or commercial platforms. The dataset is also available via https://doi.org/10.6084/m9.figshare.5904022.

The package can import data directly from excel, csv or other text format. For example, to load the data from an excel file with three separate sheets:

data<− load_excel(path=“MERGED_data.XLSX”, data_sheet=9, feature_sheet=3, sample_sheet=4)

In the ‘assayData’, the first column name is the sample IDs to match with ‘sampleData’, the other column names are metabolite IDs to match with ‘featureData’.

### 2.3 Quality control pipeline

We provided a pipeline for metabolite and sample quality control (QC) procedures with a series of functions. A minimal example is:

data_QC<− QC_pipeline(data)

In the QC pipeline, we included the following functions: remove metabolites or samples beyond a particular missing rate threshold (e.g. 0.5), detect outliers (e.g. ± 5 standard deviations) and replace outliers with missing values or winsorize outliers, and various popular methods to impute missing values (e.g. half of the minimum value, median, zero or k-nearest neighbor averaging [KNN] method). All the steps implemented in the ‘QC_pipeline’ function can be run using each individual function (e.g. ‘filter_column_missing_rate’, ‘replace_outlier’ and ‘impute’).

### 2.4 Normalization

Normalization of metabolomics data is an important step to remove systematic biases. We provide popular normalization methods: batch-based normalization, QC sample-based/nearest QC sample-based normalization and LOESS normalization. The latter three methods are useful when the measurements of internal QC samples are provided. Briefly, in the ‘batch_norm’ function, we implemented a batch-based normalization method that the raw values were divided by the median values of samples in each instrument batch to set the median value to one in each batch. ‘QCmatrix_norm’ function will use reference QC samples, such as pooled study sample reference or commercial standard QC sample to normalize raw metabolite measurement values (Ejigu *et al.*, 2013).

An example command:

data_batch_norm<− batch_norm(data)

### 2.5 Transformation

Transformation of metabolites can alter the distribution of data and is an essential step for the downstream statistical analysis. We provided the following essential transformation methods: log (natural logarithm), Pareto scale, scale and rank-based inverse normal transformation.

An example command is:

data<− transformation(data, method=“pareto_scale”)

### 2.6 Dimensional reduction

Dimensional reduction strategies on metabolites data can be used to detect batch effects, sample outliers and real biological subgroups. We included principal components analysis (PCA), manifold approximation and projection (UMAP) and t-distributed stochastic neighbor embedding methods. Figures were displayed in ggplot2 style (see the online tutorial for more details).

An example command of PCA analysis is:

data_PCA<− run_PCA(data)

### 2.7 Association analysis

Association analysis between metabolites and interested outcomes was implemented in the ‘regression’ function, supporting general linear regression, logistic regression, proportional hazards regression model, linear mixed-effects model and logistic linear mixed-effects model, with or without covariates. All the regression models can be run for selected metabolites or all metabolites in a single job with the support of parallel computing.

An example command is below:

res<− regression(object=data_batch, phenoData=df_pheno, model=“logistic”, outcome=“y”, covars=c(“age”, “sex”), ncpus=8)

### 2.8 Visualization

We provided various visualization methods for metabolomics data, including QC metrics, dimensional reduction and results visualization (displayed in the online vignette: https://xikunhan.github.io/metabolomicsR/docs/articles/Introduction.html).

### 2.9 Extension

The metabolomicsR framework is versatile and extensible, including accessibility to various analysis methods, compatibility with different measurement platforms such as LC-MS by Metabolon and nuclear magnetic resonance by Nightingale Health's platform and even adaptable to other omics data with appropriate input formats (with assay measurements, sample annotation and feature annotation). Regarding other omics, some common tasks, such as data container, transformation, regression and visualization, can be performed by our package; however, for more specific tasks, a specialized package will be more appropriate. Developers can also customize new visualization and statistical methods, or provide an interface to deploy available statistical methods based on the metabolomicsR data structure. A detailed Extension section was displayed in the online tutorial.

For example, a command to detect genuine untargeted metabolic features based on a previous method (Cao *et al.*, 2019):

res_genuMet<− genuMet_makefeature(object=data)

## 3 Discussion and conclusion

We have developed a flexible, user-friendly R package to analyze metabolomics data. The application example is based on the non-targeted mass spectrometry-based platform, however, our workflow can be readily applied to datasets from other platforms. A user-friendly streamlined workflow adapted by the research community will facilitate standardization of metabolomic analysis and ensure reproducibility of research findings.

## Funding

This work was supported by grants from the National Institutes of Health [R01 AI-148338, R01EY030088]. The content of this manuscript is solely the responsibility of the authors and does not necessarily represent the official views of the National Institutes of Health. The funding organizations were not involved in the collection, management or analysis of the data; preparation or approval of the manuscript; or decision to submit the manuscript for publication.

*Conflict of Interest*: none declared.

## Supplementary Material

vbac067_Supplementary_Data

## Data Availability

All data are incorporated into the article and its online supplementary material.
