# Supplementary material for: metabolomicsR: a streamlined workflow to analyze metabolomic data in R
Source: Bioinform Adv. 2022 Sep 16;2(1):vbac067. doi: 10.1093/bioadv/vbac067 (PMC9512519; doi:10.1093/bioadv/vbac067)
Supplement: vbac067_Supplementary_Data [file vbac067_supplementary_data.docx]

**metabolomicsR: a streamlined workflow to analyze metabolomic data in R**

Xikun Han,^1,2^ Liming Liang^1,2^

**Affiliations:**

1. Department of Epidemiology, Harvard T H Chan School of Public Health, Boston, Massachusetts, USA.
2. Program in Genetic Epidemiology and Statistical Genetics, Harvard T H Chan School of Public Health, Boston, Massachusetts, USA.

**Correspondence:**

Xikun Han, [xikun_han@hsph.harvard.edu](mailto:xikun_han@hsph.harvard.edu), and Liming Liang, [lliang@hsph.harvard.edu](mailto:lliang@hsph.harvard.edu), Harvard T H Chan School of Public Health. 655 Huntington Ave, Boston MA 02115, USA.

**Supplementary Table 1. Comparison of the main features of metabolomicsR with other metabolomics analysis software.**

| **Software** | **Main features** | **Comparison with metabolomicsR package** |
| --- | --- | --- |
| metabolomicsR | A streamlined R package for metabolomic data with a detailed online tutorial. | - |
| maplet[^1^](https://paperpile.com/c/pqXpvF/ReGRc) | A workflow to automatically record all steps, parameters and results | - Limited regression model extensions available (in metabolomicsR, association analysis for metabolites can be easily performed for a variety of models including Cox regression models, Poisson regression models, and extensible to various models (eg. negative binomial generalized linear models)); - No detailed online workflow tutorial. |
| MetaboAnalystR[^2^](https://paperpile.com/c/pqXpvF/c5r4) | Various web-based tools for metabolomic data. | - Based on web service, lack extension and streamlined workflow; - Limited regression models available (e.g. see above). |
| structToolbox[^3^](https://paperpile.com/c/pqXpvF/XiRxC) | A suite of complex class-based template and statistical analysis tools | - Complex data class definition; - no streamlined workflow; - limited regression models (see above) and normalization methods available. |

## References:

1. [Chetnik, K. *et al.* maplet: an extensible R toolbox for modular and reproducible metabolomics pipelines. *Bioinformatics* **38**, 1168–1170 (2021).](http://paperpile.com/b/pqXpvF/ReGRc)

2. [Pang, Z. *et al.* MetaboAnalyst 5.0: narrowing the gap between raw spectra and functional insights. *Nucleic Acids Res.* **49**, W388–W396 (2021).](http://paperpile.com/b/pqXpvF/c5r4)

3. [Lloyd, G. R., Jankevics, A. & Weber, R. J. M. Struct: an R/bioconductor-based framework for standardised metabolomics data analysis and beyond. *Bioinformatics* (2020) doi:](http://paperpile.com/b/pqXpvF/XiRxC)[10.1093/bioinformatics/btaa1031](http://dx.doi.org/10.1093/bioinformatics/btaa1031)[.](http://paperpile.com/b/pqXpvF/XiRxC)
